# Assessing Postoperative Handover Quality Among Nurses Across Surgical and Recovery Units: A Cross-Sectional Study

**DOI:** 10.3390/healthcare13233106

**Published:** 2025-11-28

**Authors:** Afnan M. Alotaibi, Essmat A. Mansour, Sahar M. Yakout, Amany Anwar Saeed Alabdullah

**Affiliations:** 1 Nursing Department, Huraymila General Hospital, Third Health Cluster, Riyadh 15432, Saudi Arabia; 2 Division of Medical Surgical Nursing, Department of Master in Nursing Science, College of Nursing, King Saud University, Riyadh 12372, Saudi Arabia; 3 Medical Surgical Nursing, College of Nursing, King Saud University, Riyadh 12372, Saudi Arabia; esmansour@ksu.edu.sa; 4 Maternal and Child Health Nursing, College of Nursing, King Saud University, Riyadh 12372, Saudi Arabia; szamzam@ksu.edu.sa; 5 Maternity and Pediatric Nursing, College of Nursing, Princess Nourah bint Abdulrahman University, P.O. Box 84428, Riyadh 11671, Saudi Arabia; aaalabdullah@pnu.edu.sa

**Keywords:** postoperative, quality of handover, operating theater, postanesthesia care unit, intensive care unit, wards

## Abstract

Background/Objectives: Inefficient postoperative handovers contribute to medical malpractice and care discontinuity by omitting critical patient information and compromising patient health. This study aimed to evaluate and compare the quality of postoperative nurse handovers in ORs, PACUs, ICUs, and wards across four hospitals in Jeddah, Saudi Arabia. Methods: A descriptive, cross-sectional, comparative study was conducted among postoperative care nurses across four hospitals in the second health cluster in Jeddah, Saudi Arabia. Data were collected through an online questionnaire to assess handover quality via a Handover Quality Rating Form and sociodemographic information. Data analysis was performed using SPSS v28. Results: Among the 521 nurse participants (84.1% female, M_age_ = 34.5 years), the overall postoperative handover quality was 76.8%, with handover conduct and quality scoring the highest (27.9 ± 4.8 and 17.7 ± 3.1, respectively). Female nurses demonstrated significantly higher performance in teamwork and handover circumstances, whereas older nurses demonstrated significantly better teamwork, handover conduct, and quality. Saudi and younger nurses experienced significantly higher handover circumstances. Nurses’ educational level and years of experience in the present ward were significantly correlated with handover circumstances, conduct, and quality. Handovers from the theater to recovery resulted in higher average circumstances than those from recovery to the ward. The study setting was significantly associated with handover quality. Conclusions: These findings highlight the importance of local evaluating the handover quality of nurses in various contexts, specifically considering the circumstances, conduct, and teamwork when planning implementation and developing standardized handover protocols for different departments, specialties, and healthcare settings. These results support the development of targeted training programs and unit-specific handover protocols.

## 1. Introduction

In healthcare settings, a clinical handover is an essential communication process between a sender and receiver that utilizes both verbal and written methods [1]. It is employed during patient transfers, hospital ward transitions, and staff communication within wards [2]. An effective handover is vital because it enhances communication among team members and cultivates collaboration in which healthcare personnel are well informed and actively involved in patient care planning [3]. This leads to mutual understanding and shared mental models between clinical handover and receiving professionals, especially in the operating rooms (ORs), postanesthetic care units (PACUs), and intensive care units (ICUs) [4,5]. Nevertheless, missed communication and improper patient handovers are widely recognized as causes of medical malpractice [6].

Nurse handovers occur multiple times during patient care transfers and present numerous risks of communication failure [6,7,8,9,10]. Poor nursing handover is linked to communication errors leading to patient injuries, with an estimated 80% of patients experiencing serious healthcare errors [8,9,10,11].

Postoperative handover refers to information transfer between the surgical team and postoperative care providers after surgery [12]. Surgical patients are highly vulnerable to errors because of their unstable physical condition and multiple handovers between healthcare providers [6,12,13]. Moreover, during the postoperative period, communication failure in the PACU and ICU can impact healthcare expenses and hospital length of stay and lead to mortality and morbidity [14,15]. Thus, interdepartmental collaboration among nurses is important to enhance their daily experiences, leading to information accuracy during postoperative handovers [16].

High-quality postoperative handovers are essential during the postoperative period to ensure patient safety [17,18]. However, handover quality remains variable, given the transfer of patients from the theater to the PACU, from the PACU to the ICU, and to wards that involve different specialties [19]; multiple nurses from different departments may participate in this handover process [16]. The concurrent monitoring and evaluation of patients by nurses may result in inadequate, erroneous, and inconsistent information, thereby creating communication obstacles and leading to the omission of essential details [16]. Thus, several studies have highlighted the need for a structured handover framework to enhance completeness and patient safety owing to differing expectations across specialties and departments [11,15,20].

However, to the best of our knowledge, few studies in Saudi Arabia have evaluated and compared handover quality after surgery in ORs, PACUs, ICUs, and wards [17,21]. Addressing these gaps is essential for patient safety initiatives in hospitals to provide suitable interventions. Therefore, this study aimed to evaluate and compare the quality of postoperative nurse handovers in ORs, PACUs, ICUs, and wards across four hospitals in Jeddah, Saudi Arabia.

## 2. Materials and Methods

### 2.1. Study Design, Setting and Duration

A multicenter, cross-sectional, comparative study was conducted between June 2024–August 2024, among nurses working in hospitals in the second health cluster in Jeddah, Saudi Arabia. This cluster has a capacity of 196,000 beds and covers the northern and southern regions of the city. The hospitals included King Fahad General Hospital (KFGH), King Abdullah Medical Complex (KAMC), Maternity and Children’s Specialized Hospital (MCSH), and the Eye Hospital, all under the Ministry of Health (MOH), to ensure representativeness of our research sample.

### 2.2. Study Population and Sampling

The study population included nurses from ORs, PACUs, ICUs, and wards. Participants were recruited via convenience sampling. Although convenience sampling may limit the generalizability of results, the inclusion of multiple hospitals and shifts aimed to enhance representativeness. Using the Raosoft website, the required sample size was estimated with the following parameters: 95% confidence level, z-score 1.96, 5% margin of error, and 50% population proportion. Based on these parameters, the estimated sample size was 965 nurses. The hospitals included in the study were (KFGH, *n* = 408), KAMC, *n* = 409), the Eye Hospital (*n* = 46), and MCSH (*n* = 102). Of this population, 521 nurses ultimately completed the questionnaire.

### 2.3. Eligibility Criteria

The inclusion criteria were nurses employed in critical care settings, ICUs, ORs, and general wards (in this study, general wards and intensive care units refer to surgical or general, maternity, and pediatric units) who provide care and services to postoperative patients, specifically those who transfer or receive postoperative endorsement sheets, irrespective of their age, nationality, or gender. They should have at least six months of experience in the department, be able to complete the questionnaire, and be willing to participate. Exclusion criteria included head nurses and nurses not involved in direct patient care, anesthesia technicians, and nurses working in noncritical care units who did not participate in endorsements.

### 2.4. Study Tool

A structured English questionnaire comprising two sections was used. The first section collected sociodemographic data, which included nurses’ age, gender, nationality, marital status, educational level, years of experience, department, current nursing role, and extent of experience based on the current unit. The second section comprised the Handover Quality Rating Form (HQRF) developed by Manser et al. (2010, 2013) [22,23]. This tool comprises 21 items divided into four subscales: handover circumstances (four items), handover conduct (eight items), teamwork (four items), and handover quality (five items). The items are rated on a 4-point Likert scale, where a score of 1 = disagree, 2 = partially disagree, 3 = partially agree, and 4 = agree. In this study, the reliability and validity were assessed via Cronbach’s alpha coefficient, which was 0.76 for “handover circumstances,” 0.767 for “handover conduct,” 0.624 for “teamwork” and 0.783 for “handover quality.”

In this study, Cronbach alphas for four subscales were 0.76, 0.767, 0.624, 0.783, respectively. The instrument was previously validated and conducted in English, which is widely recognized as a healthcare professional language in Saudi Arabia.

### 2.5. Data Collection

The researchers visited the potential participants in the selected study settings, introduced themselves, and explained the purpose of the study. Participant nurses were required to fill in an anonymous, self-administered questionnaire via the online survey platform Google Forms (Google LLC, USA) located in Mountain View, California, United States. Data were collected daily and from those on night and day shifts as well as from weekday and weekend shifts. At the end of the endorsements, a quality rating form was completed by each nurse who handed over the patients and the nurse who took responsibility for them. The data collection process was conducted after obtaining IRB approval and institutional permission from all four hospitals. Potential participants were approached by a research assistant during non-clinical times (breaks/meetings) and provided with an Information sheet detailing the study’s purpose and voluntary nature. Informed consent was implied by the voluntary act of completing the anonymous questionnaire. To ensure anonymity and minimize social desirability bias, the questionnaire did not request any personal identifiers. Completed surveys were placed in locked, non-transparent collection boxes in a neutral location within each unit before being transported securely by the research team for data entry.

### 2.6. Pilot Study

A pilot study was initially administered to evaluate the clarity, applicability, precision, and time required to complete the questionnaire. The sample included 15 nurses who met the same eligibility criteria and accounted for 10% of the total sample size. The same participants were not included in the main study to eliminate the risk of priming effects and maintain the main study. Finally, the questionnaire was completed without any modifications made to the core content.

### 2.7. Ethical Considerations

Study approval was obtained from the scientific research ethics committee of the King Saud University (approval no. KSU-HE-24-445) and the institutional review board of Jeddah, MOH (IRB log number A01959). All participants provided informed consent before responding to the questionnaire to ensure voluntary participation. The participants were informed that they could withdraw from the study at any time without any conflict or harm. The responses provided by the nurses were coded anonymously with codes and numbers to ensure confidentiality.

### 2.8. Data Analysis

Data were analyzed using the Statistical Package for the Social Sciences (SPSS, version 28). Normality tests were conducted using Kolmogorov’s test. Numerical variables are presented as means, standard deviations (SDs), and medians (ranges), and categorical variables are presented as frequencies and percentages. The nonparametric Mann–Whitney U test was used to measure the relationship between two numerical groups, whereas the Kruskal–Wallis test was used to examine the correlation among three independent groups. A post hoc test with Bonferroni correction was performed to determine statistical significance, and a threshold value of 0.05 was applied. Statistical significance was set at *p* < 0.05.

## 3. Results

Among the 521 participant nurses, the majority were female (84.1%), Saudi nationals (86.6%), and married (62.8%); their mean age was 34.5 years, with 60.3% in the 30–40 years age group. More than half (57.8%) had a bachelor’s degree, 76% had >2 years of experience, 56.2% were working in the wards, 41.1% had 6–10 years of experience in their present unit, and most of them (81.8%) were currently staff nurses (Table 1).

Table 2 presents the frequency distribution of the nurses’ responses regarding various aspects of handovers. Regarding the circumstances of the handover, nearly more than half of nurses (67.6%) agreed that handovers did not involve high levels of uncertainty. A total of 53.3% and 56.3% of nurses agreed that the person handing over and the person taking on responsibility for the patient were not under time pressure, respectively. 

Regarding the handover conduct, majority of the nurses agreed that the handover followed a logical structure (88.4%), all relevant information was selected and communicated (90.4%), priorities for further treatment were addressed (88.3%), the person handing over the patient communicated their assessment of the patient (90.4%), and possible risks and complications were discussed (72%). 

With respect to teamwork, the majority of the nurses agreed that the team jointly ensured that the handover was complete (78.3%), and 73.1% agreed that questions and ambiguities were resolved. Regarding handover quality, more than three-fourths (78.7%) of the participants agreed that the documentation was complete, and 77% agreed that the patient’s experience was considered carefully during the handover. Overall, 76.8% of the nurses agreed that the handover quality was very high. Additional data are provided in the Supplementary Materials Table S1.

As shown in Table 3, the highest-rated aspect of handover quality among the nurses was handover conduct, with a mean score of 27.9 ± 4.8. By contrast, handover circumstances received the lowest mean score of 10.4 ± 3.9.

Table 4 presents the correlations between the sociodemographic factors and the four domains of handovers are presented. Handover circumstances were significantly related to age (*p* = 0.004), gender (*p* = 0.046), nationality (*p* = 0.033), educational level (*p* = 0.021), and years of experience in the present unit (*p* < 0.001). Moreover, significantly better handover circumstances were found among nurses in the theater recovery department (*p* = 0.023) and among transferring team members than among receiving team members (*p* < 0.001).

With regard to the handover conduct, significant correlation was found with age (*p* = 0.011), nationality (*p* = 0.008), educational level (*p* = 0.007), total years of experience (*p* < 0.001), and years of experience in the present unit (*p* < 0.001).

Regarding teamwork, a statistically significant relationship was found between age (*p* = 0.022) and gender (*p* = 0.004). Additionally, the mean score was significantly higher among transferring team members than among receiving team members (*p* < 0.001).

With regard to handover quality, significant associations were observed between age (*p* = 0.007), nationality (*p* = 0.011), educational level (*p* = 0.001), and years of experience in the present unit (*p* < 0.001).

Table 5 shows the differences in the quality of postoperative handovers in different study hospitals in Jeddah. There was a significant relationship between the study setting and the circumstances of handovers, with an overall *p*-value < 0.001. After conducting a pairwise comparison, KFGH had a significantly lower average than the other three hospitals (adjusted *p*-value = 0.002, <0.001, 0.004, respectively). The Eye Hospital had significantly higher average handover circumstances than KAMC and MCSH (adjusted *p*-value = 0.013, 0.002, respectively).

Similarly, handover conduct showed a significant relationship with hospital settings, with an overall *p*-value of <0.001. After performing a pairwise comparison, KAMC had a significantly lower average of handover conduct than KFGH and the Eye Hospital (adjusted *p*-values = <0.001 and <0.001, respectively). Additionally, MCSH had a significantly lower average of handover conduct than KFGH and Eye Hospital (adjusted *p*-value = <0.001 and 0.002, respectively).

There was a statistically significant relationship between the study setting and teamwork, with an overall *p*-value < 0.001. After conducting a pairwise comparison, the Eye Hospital showed significantly higher average teamwork than KAMC, KFGH, and MCSH (adjusted *p*-values = <0.001, 0.017, 0.044, respectively).

There was a statistically significant relationship between the study setting and handover quality with an overall *p*-value < 0.001. After performing a pairwise comparison, KFGH had a significantly higher average handover quality than the Eye Hospital, KAMC, and MCSH (adjusted *p*-value = 0.003, <0.001, <0.001, respectively). Moreover, KAMC had a lower average handover quality than the Eye Hospital (adjusted *p*-value = 0.002).

## 4. Discussion

In this study, the overall handover experience of nurses in four hospitals in Jeddah, Saudi Arabia, was high at 76.8%. Among the four domains examined, the mean score for handover conduct was found to be the highest, with approximately more than three-quarters of the nurses reporting following a logically structured handover and discussing potential risks and complications. More than three-quarters of the participants reported using documentation, communicating patient information, therapeutics, and essential assessments. These findings suggest that handover conduct is generally well-structured and can be compared to a cross-sectional study by Leonardsen et al. (2019) [24] on the quality of OR and PACU postoperative handovers in Norway, which reported that over two-thirds of the participants adhered to the logical structure and that all relevant patient information was communicated. By contrast, less than half the staff utilized patient documents during patient care, resulting in staff disruptions and a lack of attempts to reduce interruptions. Additionally, nurses’ transferring more than required information prolonged the handover time, which contributed to the overall low quality of the handover. Previous studies conducted in Germany and southern Ethiopia that evaluated handovers from ORs to the PACU and OR/PACU to ICU settings revealed that less than half the pertinent information that was documented, which encompassed diagnostics and pain treatment, was accurately conveyed [12,19]. The presence of a supporting framework, less disruption, and the duration of the handover have been found to be correlated with essential information transfer [19,24,25].

With regard to the circumstances of the handover, more than one-third of the participants agreed that the person handing over the patient and the receiver did not face any time constraints or pressure and that the cases handed over were not highly complex; more than half responded that the cases did not involve high uncertainty. These findings are consistent with those of earlier studies in Saudi Arabia and Norway, in which more than half the nurses reported high-quality handovers. However, the handover technique continues to be influenced by the complexity of the patient’s health status and time pressure during the handover [17,18]. Nevertheless, a previous study noted that handover processes were not significantly altered, even in complex and high-risk pregnancy cases, when in congruence with the best-practice environment [26]. Conversely, a study conducted in Australian hospitals revealed that disorganized and inconsistent handover processes adapted to each patient’s needs led to patient complexity and time restrictions that compromised the quality of nurses’ handovers [27]. This highlights variations in postoperative transfer items and tasks that could be attributed to low checklist compliance, situational circumstances, and patient conditions [18].

Most of the items under teamwork were rated highly. The nurses reported high agreement (91.5%) regarding jointly ensuring that the handover was complete, and more than three-quarters reported resolving questions and ambiguities. Moreover, 75.4% and 67.6% reported that it was easy to establish contact before the handover, and there was no tension between teams during the handover [24]. In an earlier study, agreement with establishing good contact at the beginning of a handover and resolving ambiguities was reported among more than three-fourths of the study participants, and more than two-fourths agreed that the team jointly ensured that the handover was complete [24]. By contrast, the findings from some studies did not support positive teamwork; In New Zealand, Eton (2020) was unable to demonstrate effective handover among team members, primarily due to the inability of PACU nurses to pose inquiries because of multitasking and OR nurses’ rapid return to the theater under tension [25]. Another study proposed that flexibility in handovers, reporting, and teamwork improved the quality of postoperative handovers [28].

Participants evaluated handover quality as high. More than three-quarters of the nurses reported that the documentation was complete and considered the patient’s experience. These findings indicate that most nurses in critical departments have clear experience and knowledge of handling handovers [21]. These results differ from other studies in which the overall quality of postoperative nursing and shift handover scores were higher than those reported in other international hospitals [12,18,29,30] and lower than those reported in a local study in Saudi Arabia [17]. Research has described handover as a multidimensional phenomenon affected by factors such as high staffing, cognitive capacity, effective communication, and non-technical skills among healthcare providers [12,21].

Regarding the correlation between personal demographics and handover quality, nurses aged >40 years had significantly higher mean scores for handover conduct, teamwork, and quality than their younger counterparts. Nurses’ professional experience increases with age, which helps them promptly identify patients by sharing information about their health issues and ensuring that decisions are made quickly and effectively. By contrast, newly graduated nurses encounter challenges and are less experienced in performing appropriate clinical handovers [20,31].

Additionally, female nurses scored significantly higher on handover circumstances and teamwork than male nurses did. This could be attributed to the greater proportion of female nurses in the study and their comprehensive and collaborative communication skills. Similarly, Hashish et al. reported that female nurses scored higher on handover quality, highlighting their different roles and dominance in this domain [21]. Inconsistent with our findings, another study found no significant difference between male and female nurses in terms of factors that affect postoperative handovers [32].

In contrast to the findings of previous studies, the present study indicates that Saudi nationality was significantly associated with better handover circumstances, conduct, and quality among nurses. One explanation is that the nationality association might be influenced by nurse-tailored training programs aligned with the local clinical protocols in Saudi Arabia for new graduates, which can help them overcome the transition in care challenges [33,34]. Saudi students are willing to improve their knowledge and practices based on the learning environment of Saudi Arabian hospitals. Moreover, in Saudi Arabia, predominantly expatriate non-Saudi nursing staff may face communication limitations and linguistic and religious differences. However, these interpretations remain hypothetical and should be approached cautiously, and the results require further qualitative investigation into communication strategies, which could provide a better consideration of individual practices regarding the quality of handovers in Saudi Arabia [21].

A disparity in education scores was also found, which may be attributed to significant variances in the educational background of nurses from diplomas, bachelor’s degrees in nursing, to specialist nurses [35,36]. Nurses with more than two years of total experience had significantly higher scores for handover conduct. This may be due to the inability of less experienced nurses to provide adequate information. Compared with more experienced nurses, newly graduated nurses are less predisposed to seeking inconsistencies in the communication process [20,37]. By contrast, a South Korean study reported that nurses with 12 months of work experience exhibited superior handover evaluations [36]. This finding highlights the necessity of paying more attention to providing clear handover guidelines and continuous evaluation of nurse work.

In terms of experience in the present unit, 6–10 years was significantly associated with excellence in conduct and quality of the postoperative handover. This implies that increased practice contributes to greater familiarity with handover policies and department protocols. In alignment with the findings of the present study, experienced OR nurses emphasize safety, enhanced quality, and management more than their younger and less-experienced counterparts [38].

Compared to receiving team members, transferring team members showed significantly better handover circumstances and teamwork. These disparities could be attributed to different anticipatory approaches to information sharing. Transfer nurses tend to have high handover quality scores because of their various responsibilities [18].

In general, the findings of this study revealed significant associations between the different hospital settings and the four domains of handovers. Notably, the Eye Hospital scored significantly higher in handover circumstances compared to the other three hospitals, suggesting that the nature of the setting, environment, and institutional practices and policies may affect the care process. The Eye Hospital also scored significantly higher on teamwork. This could be attributed to the hospitals’ superior handover circumstances. Evaluating differences in the quality of postoperative handovers across study settings is crucial for developing standardized and effective assessment programs that directly enhance the transfer of patient care.

### 4.1. Implications for Nursing Practice and Research

It is imperative that nurse researchers and clinical postoperative nurses collaborate actively to develop effective handover processes. Handover should be continuously evaluated and refined to enhance the quality of postoperative healthcare. Nurses in hospitals can implement handover beyond information transfer through work with multidisciplinary teamwork and transparent transfer of care. These cooperation, circumstances, education, and technology integration can be applied when assessing handover practices to support consistency and high-quality care across healthcare settings.

This research enhances the existing research in postoperative handover quality. Healthcare organizations should develop targeted interventions to establish nationally standardized protocols and training programs to guide the development of training and internship programs. Moreover, consistent education in academic institutions to integrate educational programs into curricula can strengthen the future nurses to acquire the necessary communication, critical thinking, and decision-making skills to perform efficient and effective handovers.

### 4.2. Strength and Limitation

Previous studies have evaluated handover quality in single-setting areas in Saudi Arabia, whereas our study focused on assessing and comparing handover quality over a specific period in multiple settings. Unlike previous research, this study integrated various departments and settings simultaneously with diverse levels of quality, allowing for an assessment of how different institutional environments affect handover practices.

However, this study has some limitations that merit consideration. This study’s cross-sectional design limited its ability to establish causal relationships and the generalizability of the result. A longitudinal study would provide a better understanding of the evolution of these factors over time. Convenience sampling is more prone to selection bias, compromises external validity, and limits representative of all nurses in the second health cluster of Jeddah. Self-report questionnaires might have introduced response and social desirability bias, as participants provided answers they perceived as more proficient. The study mentioned differences in handover quality across different hospital settings; however, it did not compare how specific departmental challenges (e.g., ICU vs. ward vs. PACU) affect handover quality.

### 4.3. Recommendations and Future Directions

Future research could be replicated in multiple sectors within the Saudi Arabian MOH, emphasizing that nursing schools integrate structured handover training and educational programs into their curricula. Nursing administration at the MOH and hospital health clusters can develop interventions, resulting in nationally standardized handover protocols across healthcare facilities. It is also crucial to provide internship training programs within these protocols. For nursing practice, evidence-based recommendations for improving nurses’ handover quality through knowledge and standardization can be made. Hospital nurses can implement handovers beyond information transfer through multidisciplinary teamwork and transparent transfer of care to improve postoperative handovers and enhance patient safety. Cooperation, circumstances, education, and technological integration should also be considered.

## 5. Conclusions

In conclusion, this study revealed high-quality postoperative handovers among nurses in the ORs, PACUs, ICUs, and wards in four hospitals in Saudi Arabia, with notable differences in handover quality with nurses’ sociodemographic and compared settings. The findings emphasize the need for context-specific handover guidelines and unit specific handover protocol for potential national policy to mitigate the negative impacts of disparities in handovers, especially among post-surgical nurses, to promote early assessment and reduce the low quality of handovers.

## Figures and Tables

**Table 1:** Study participants’ sociodemographic characteristics.

Characteristics	Total (*n* = 521)
Age (in years)	
Mean ± SD	34.5 ± 6.7
Median (range)	34 (20–60)
Age-groups	
20–30 years	143 (27.4%)
30–40 years	314 (60.3%)
>40 years	64 (12.3%)
Gender	
Female	438 (84.1%)
Male	83 (15.9%)
Nationality	
Non-Saudi	70 (13.4%)
Saudi	451 (86.6%)
Marital status	
Not married	194 (37.2%)
Married	327 (62.8%)
Education level	
Bachelor’s	301 (57.8%)
Diploma	188 (36.1%)
Master’s or higher	32 (6.1%)
Total years of experience	
<1 years	31 (6%)
1–2 years	94 (18%)
>2 years	396 (76%)
Department	
ICU	58 (11.1%)
Maternity ICU	7 (1.3%)
Maternity Ward	27 (5.2%)
Pediatric ICU	18 (3.5%)
Pediatric Ward	5 (1.0%)
Recovery Room (PACU)	50 (9.6%)
Theater (OR)	63 (12.1%)
Ward	293 (56.2%)
Years of experience in the present unit	
1–5 years	199 (38.2%)
6–10 years	214 (41.1%)
>10 years	108 (20.7%)
Current nursing role (position)	
Charge nurse	95 (18.2%)
Staff Nurse	426 (81.8%)
Hand over (from-to)	
Recovery-Pediatric Ward	17 (3.3%)
Recovery-Cardiac ICU	18 (3.5%)
Recovery-General ICU	44 (8.4%)
Recovery-Maternity ICU	5 (1%)
Recovery-Maternity Ward	24 (29.6%)
Recovery-Pediatric ICU	14 (2.7%)
Recovery-Ward	328 (63%)
Theater-Recovery	71 (13.6%)
Rater	
Receiving team member: Nurse	310 (59.5%)
Transferring team member: Nurse	211 (40.5%)
Setting sample size	
King Fahad General Hospital (KFGH)	199 (38%)
King Abdullah Medical Complex (KAMC)	199 (38%)
Maternity and Children’s Specialized Hospital (MCSH)	81 (15.5%)
Eye Hospital	42 (8%)

SD; standard deviation, ICU; Intensive Care Unit, PACU; Post Anesthesia Care Unit, OR; operating room.

**Table 2:** Frequency distribution of nurses’ responses on each item of Handover Quality Rating Form (HQRF).

	Agreement (Partially Agree + Agree)
**Handover Circumstances**	
The person handing over the patient was not under time pressure.	278 (53.3%)
The person taking on the responsibility of the patient was not under time pressure.	293 (56.3%)
The case that was handed over was not of high complexity.	267 (51.2%)
The case that was handed over did not involve high uncertainty.	352 (67.6%)
**Handover Conduct**	
The handover followed a logical structure.	461 (88.4%)
The person handling the patient used available documentation to structure the handover.	480 (92.1%)
Enough time was allowed for the handover.	325 (62.4%)
In case of interpretation, attempts were made to minimize interruptions during the handover.	444 (85.2%)
All relevant information was selected and communicated.	471 (90.4%)
Priorities for further treatment were addressed.	460 (88.3%)
The person handing over the patient clearly communicated their assessment of the patient.	471 (90.4%)
Possible risks and complications were discussed.	456 (87.5%)
**Teamwork**	
It was easy to establish good contact at the beginning of the handover.	393 (75.4%)
There was no tension between the team during the handover.	352 (67.6%)
Questions and ambiguities were resolved.	474 (91.0%)
The team jointly ensured that the handover was complete.	477 (91.5%)
**Handover quality**	
Documentation was complete.	480 (92.1%)
Not too much information was given.	432 (82.9%)
Not too much information was asked for.	332 (63.7%)
The patient’s experience was considered carefully during the handover (respect).	401 (77%)
Overall, the quality of this handover was very high.	400 (76.8%)

**Table 3:** Level of postoperative handover quality.

Characteristics	Total (*n* = 521)
Handover circumstances	
Mean ± SD	10.4 ± 3.9
Median (range)	10 (4–16)
Handover conduct	
Mean ± SD	27.9 ± 4.8
Median (range)	29 (8–32)
Teamwork	
Mean ± SD	13.4 ± 2.1
Median (range)	13 (4–16)
Handover quality	
Mean ± SD	17.7 ± 3.1
Median (range)	20 (5–20)

Data are presented as means ± standard deviations (SD), median, and range.

**Table 4:** Correlation between personal demographics with handover circumstances, conduct, quality, and teamwork.

	Handover Circumstances		Handover Conduct		Teamwork		Handover Quality	
Characteristics	Mean ± SD	*p*-Value	Mean ± SD	*p*-Value	Mean ± SD	*p*-Value	Mean ± SD	*p*-Value
**Age Groups (years)**		0.004 *		0.011 *		0.022 *		0.007 *
20–30	11.4 ± 3.8		28.1 ± 4.3		13.5 ± 2.2		17.9 ± 3.11	
30–40	10.1 ± 3.9		27.6 ± 4.9		13.2 ± 2.2		17.4 ± 3.1	
>40	10.4 ± 4.0		29.3 ± 3.7		14.0 ± 1.6		18.6 ± 2.2	
**Gender**		0.046 *		0.583		0.004 *		0.758
Female	10.6 ± 4.0		28.1 ± 4.5		13.5 ± 2.2		17.8 ± 3.08	
Male	9.7 ± 3.6		27.3 ± 5.3		12.9 ± 2.1		17.7 ± 3.13	
**Nationality**		0.033 *		0.008 *		0.136		0.011 *
Non-Saudi	9.6 ± 4.2		27.2 ± 4.3		13.0 ± 2.2		17.1 ± 3.3	
Saudi	10.6 ± 3.9		28.1 ± 4.7		13.5 ± 2.2		17.8 ± 3.1	
**Marital status**		0.074		0.708		0.484		0.269
Not married	10.9 ± 3.9		27.8 ± 4.8		13.4 ± 2.4		17.8 ± 3.1	
Married	10.2 ± 3.9		28 ± 4.6		13.4 ± 2.1		17.6 ± 3.0	
**Education level**		0.021 *		0.007 *		0.498		0.001 *
Bachelor’s	10.6 ± 3.9		28.1 ± 4.1		13.4 ± 2.1		17.8 ± 2.8	
Diploma	10.1 ± 3.9		28 ± 5.4		13.4 ± 2.2		17.9 ± 3.2	
Master’s or higher	12.2 ± 3.5		26.4 ± 4.3		12.9 ± 2.5		15.7 ± 4.1	
**Total years of experience**		0.307		<0.001 *		0.096		0.36
<1 years	11.2 ± 3.7		24.6 ± 5.7		12.4 ± 3.4		16.6 ± 4.2	
1–2 years	10.9 ± 3.3		27.3 ± 5.1		13.1 ± 2.4		17.6 ± 3.3	
>2 years	10.3 ± 4.1		28.4 ± 4.4		13.6 ± 2.0		17.8 ± 2.9	
**Department**								
ICU	9.9 ± 3.9	0.052	28.5 ± 4.1	0.056	13.3 ± 2.1	0.197	17.7 ± 3.4	0.283
Maternity ICU	9.9 ± 4.0		29.6 ± 2.5		13.9 ± 1.1		17.7 ± 2.1	
Maternity Ward	10.0 ± 4.0		25.7 ± 6.5		12.5 ± 2.5		16.8 ± 4.3	
Pediatric ICU	9.5 ± 4.1		26.9 ± 3.8		13.0 ± 2.3		17 ± 2.3	
Pediatric Ward	10.2 ± 4.6		28.6 ± 3.7		13.9 ± 1.9		18.6 ± 2.1	
Recovery Room (PACU)	11.8 ± 3.9		28.3 ± 4.1		13.8 ± 2.6		17.6 ± 3.4	
Theater (OR)	11.6 ± 3.6		27 ± 4.9		13.5 ± 2.2		17.8 ± 2.5	
Ward	10.3 ± 3.8		28.3 ± 4.6		13.5 ± 2.1		17.8 ± 2.9	
**Years of experience in the present unit**		<0.001 *		<0.001 *				
1–5 years	11.6 ± 3.5		27.2 ± 4.4		13.3 ± 2.4	0.785	17.1 ± 3.1	<0.001 *
6–10 years	9.6 ± 3.8		28.9 ± 4.6		13.5 ± 1.9		18.3 ± 2.9	
>10 years	10.2 ± 4.4		27.5 ± 4.9		13.4 ± 2.2		17.8 ± 3.2	
**Current nursing role (position)**								
Charge nurse	10.2 ± 4.4	0.507	27.5 ± 4.8	0.104	13.1 ± 2.6	0.471	17.6 ± 3.5	0.894
Staff Nurse	10.6 ± 3.8		28 ± 4.6		13.5 ± 2.0		17.8 ± 2.9	
**Hand over (from-to)**		0.023 *		0.053		0.462		0.407
Recovery-Pediatric Ward	10.3 ± 4.6		28.7 ± 3.5		13.7 ± 2.1		18.3 ± 2.61	
Recovery-Cardiac ICU	9.9 ± 3.6		29.3 ± 3.9		13.4 ± 3.1		20 ± 9.0	
Recovery-General ICU	10.5 ± 4.1		27.8 ± 4.5		13.2 ± 2.2		17.3 ± 3.6	
Recovery-Maternity ICU	9.0 ± 4.6		27.3 ± 4.7		13.6 ± 2.7		18.4 ± 1.9	
Recovery-Maternity Ward	10.9 ± 4.0		26.1 ± 5.6		13.2 ± 2.4		17.02 ± 3.8	
Recovery-Pediatric ICU	9.3 ± 4.0		28.3 ± 2.2		13.3 ± 1.8		17.5 ± 2.1	
Recovery-Ward	10.3 ± 3.8		28.3 ± 4.6		13.3 ± 2.0		17.8 ± 3.04	
Theater-Recovery	12.0 ± 3.4		27.2 ± 5.0		13.7 ± 2.4		17.9 ± 2.8	
**Rater**		<0.001 *		0.033		<0.001 *		0.09
Receiving team member: Nurse	9.7 ± 3.7		28.2 ± 4.7		13.1 ± 2.1		17.7 ± 3.31	
Transferring team member: Nurse	11.7 ± 3.9		27.6 ± 4.6		13.8 ± 2.2		17.7 ± 2.7	

SD; standard deviation, ICU; Intensive Care, Unit PACU; Post Anesthesia Care Unit. * Significant *p*-value (*p* < 0.05).

**Table 5:** Difference in quality of postoperative handover in different study hospitals.

Characteristics	Eye Hospital (*n* = 42)	King Abdullah Medical Complex (*n* = 199)	King Fahad General Hospital (*n* = 199)	Maternity and Children’s Specialized Hospital (*n* = 81)	Test	*p*-Value
Handover circumstances						
Mean ± SD	13.3 ± 3.4	11.4 ± 3.6	8.9 ± 3.6	10.7 ± 4.1	64.1	<0.001 *
Median (range)	15 (4–16)	12 (4–16)	7 (4–16)	11 (4–16)		
Handover conduct						
Mean ± SD	29.1 ± 3.5	26 ± 4.8	30.3 ± 3.3	26.4 ± 5.0	144.3	<0.001 *
Median (range)	29 (18–32)	27 (8–32)	32 (15–32)	28 (8–32)		
Teamwork						
Mean ± SD	14.5 ± 1.9	13 ± 2.4	13.6 ± 1.6	13.3 ± 2.6	18.1	<0.001 *
Median (range)	15 (8–16)	13 (4–16)	13 (7–16)	13 (4–16)		
Handover quality						
Mean ± SD	18.3 ± 2.2	16.4 ± 3.4	19.2 ± 1.9	17.15 ± 3.18	124.4	<0.001 *
Median (range)	20 (14–20)	17 (5–20)	20 (9–20)	18 (5–20)		

SD; standard deviation, * Significant *p*-value (*p* < 0.05).

## Data Availability

The data presented in this study are available upon request from the corresponding author due to privacy reasons.

## Supplementary Materials

The following supporting information can be downloaded at https://www.mdpi.com/article/10.3390/healthcare13233106/s1, Table S1: Frequency distribution of nurses’ responses on each item of Handover Quality Rating Form (HQRF).
