# Supplementary material for: Assessing Postoperative Handover Quality Among Nurses Across Surgical and Recovery Units: A Cross-Sectional Study
Source: Healthcare (Basel). 2025 Nov 28;13(23):3106. doi: 10.3390/healthcare13233106 (PMC12692589; doi:10.3390/healthcare13233106)
Supplement: Supplementary file 1 [file healthcare-13-03106-s001.zip › healthcare-3951747-supplementary.pdf]

Table S1. Frequency distribution of nurses' responses on each item of Handover Quality Rating Form (HQRF)

	Disagree	Partially disagree	Partially agree	Agree
**Handover Circumstances**				
The person handing over the patient was not under time pressure.	201 (38.6%)	42 (8.1%)	92 (17.7%)	186 (35.7%)
The person taking on the responsibility of the patient was not under time pressure.	193 (37%)	35 (6.7%)	110 (21.1%)	183 (35.1%)
The case that was handed over was not of high complexity.	193 (37%)	61 (11.7%)	99 (19%)	168 (32.2%)
The case that was handed over did not involve high uncertainty.	111 (21.3%)	58 (11.1%)	83 (15.9%)	269 (51.6%)
**Handover Conduct**				
The handover followed a logical structure.	31 (6%)	29 (5.6%)	77 (14.8%)	384 (73.7%)
The person handling the patient used available documentation to structure the handover.	16 (3.1%)	25 (4.8%)	75 (14.4%)	405 (77.7%)
Enough time was allowed for the handover.	124 (23.8%)	72 (13.8%)	83 (15.9%)	242 (46.4%)
In case of interpretation, attempts were made to minimize interruptions during the handover.	34 (6.5%)	43 (8.3%)	85 (16.3%)	359 (68.9%)
All relevant information was selected and communicated.	18 (3.5%)	32 (6.1%)	77 (14.8%)	394 (75.6%)
Priorities for further treatment were addressed.	26 (5.0)	35 (6.7%)	66 (12.7%)	394 (75.6%)
The person handing over the patient clearly communicated their assessment of the patient.	20 (3.8%)	30 (5.8%)	75 (14.4%)	396 (76%)
Possible risks and complications were discussed.	27 (5.2%)	38 (7.3%)	81 (15.5%)	375 (72%)
**Teamwork**				
It was easy to establish good contact at the beginning of the handover.	106 (20.3%)	22 (4.2%)	82 (15.7%)	311 (59.7%)
There was no tension between the team during the handover.	113 (21.7%)	56 (10.7%)	77 (14.8%)	275 (52.8%)
Questions and ambiguities were resolved.	20 (3.8%)	27 (5.2%)	93 (17.9%)	381 (73.1%)

The team jointly ensured that the handover was complete.	18 (3.5%)	26 (5%)	69 (13.2%)	408 (78.3%)
**Handover quality**				
Documentation was complete.	18 (3.5%)	23 (4.4%)	70 (13.4%)	410 (78.7%)
Not too much information was given.	42 (8.1%)	47 (9%)	87 (16.7%)	345 (66.2%)
Not too much information was asked for.	45 (8.6%)	61 (11.7%)	83 (15.9%)	332 (63.7%)
The patient's experience was considered carefully during the handover (respect).	24 (4.6%)	22 (4.2%)	74 (14.2%)	401 (77%)
Overall, the quality of this handover was very high.	16 (3.1%)	17 (3.3%)	88 (16.9%)	400 (76.8%)
